# 

**Published:** 1884-12

**Authors:** 


### CONTENTS.

### *MISCELLANEOUS.*

| | PAGE |
|---|---|
| Hahnemann and Cholera Microbes. R. E. Dudgeon, M. D., . . | 335 |
| Hahnemann's Homœopathic Formula. A. Lippe, M. D., . . | 337 |
| Then and Now: What has Caused the Change. P. P. Wells, M. D., | 339 |
| A Condemnation of Specialism in Medicine, . . . . . . | 346 |
| Joslin on Cholera, . . . . . . . . . . . . . | 346 |
| The Symptom Which Indicates the Remedy. T. L. Brown, M. D., . | 347 |
| Lectures on Materia Medica, . . . . . . . . . . | 352 |
| Notes on Aceticum Acidum, . . . . . . . . . . | 352 |
| In Memoriam: George Hosfeld, M. D., . . . . . . . . | 354 |

### *CLINICAL BUREAU.*

| | |
|---|---|
| A Case of Hay Fever. W. S. Gee, M. D., . . . . . . | 355 |
| What I Know About Phytolacca. Wm. Jefferson Guernsey, M. D., . | 357 |
| Diphtheria—Phytolacca. Walter M. James, M. D., . . . . . | 359 |
| Clinical Notes. J. T. Kent, M. D., . . . . . . . . . | 360 |

### *BOOK NOTICES.*

| | |
|---|---|
| The Treatment of Uterine Displacements. William Eggert: Duncan Bros., . . . . . . . . . . . . . . . | 361 |

### *NOTES AND NOTICES.*

| | |
|---|---|
| Removal.—Dr. C. S. Fahnstock.—Laying a Corner-stone.—A New Journal.—Lippe's Repertory.—Knowledge Regarding Cholera.—Nil, . . . . . . . . . . . . . . . | 362 |

